# Oxidative Damage and Antioxidative Therapy in Systemic Sclerosis

**DOI:** 10.1155/2014/389582

**Published:** 2014-09-08

**Authors:** Bogna Grygiel-Górniak, Mariusz Puszczewicz

**Affiliations:** Department of Rheumatology and Internal Medicine, Poznan University of Medical Sciences, 28 Czerwca Street 135/147, 61-545, Poznan, Poland

## Abstract

Systemic sclerosis (SSc) is an autoimmune connective tissue disorder of unknown etiology. This disease is characterized by a large variety of clinical patterns, which include the fibrosis of skin and visceral organs causing a variety of clinical manifestations. Genetic and environmental factors participate in the etiology of this disease; however, recently many studies underline the oxidative background influencing the course and complications of this disease. Reactive oxygen species (ROS) synthesized in SSc can mediate extra- and intracellular oxidative processes affecting endothelial cells and fibroblasts. The estimation of prooxidative markers in the pathogenesis of SSc can enable the identification of useful markers for disease activity and, thus, may help in planning appropriate therapy focusing on the fibrotic or vascular pattern. Recently, many attempts have been made to find antioxidative molecules (nutritional and pharmacological) reducing the prooxidant state in a variety of cells—mainly in endothelium and proliferating fibroblasts. This paper presents both the background of oxidative stress processes in systemic sclerosis mediated by different mechanisms and the evidence suggesting which of the dietary and pharmacological antioxidants can be used as therapeutic targets for this disease.

## 1. Introduction

Systemic sclerosis is an autoimmune disease characterized by vascular hyperreactivity and fibrosis of skin and visceral organs [1, 2]. The degree of skin fibrosis, immunological profile, and microvascular dysfunction determines the clinical classification of the disease, which includes limited (lSSc) and diffuse (dSSc) cutaneous SSc [3, 4]. An excessive fibrosis is the most characteristic pathological manifestation of SSc, particularly evident in the diffuse cutaneous form of this disease [3]. The overproduction of the extracellular matrix by fibroblasts and myofibroblasts is responsible for increased collagen synthesis and its deposition in the skin, lungs, heart, gastrointestinal tract, kidney, tendons, and ligaments [2, 3]. Increased fibrosis causes organ dysfunction and is responsible, to a large extent, for morbidity and mortality in SSc.

**Figure 1:**
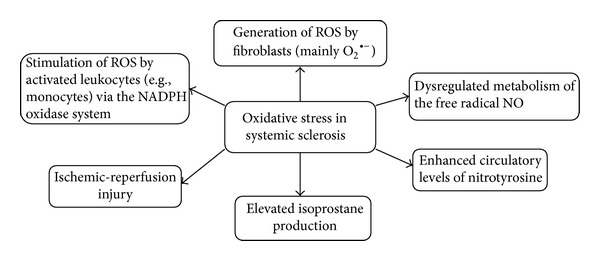
The main mechanisms of oxidative stress in systemic sclerosis. NADPH oxidase: nicotinamide adenine dinucleotide phosphate-oxidase; oxLDL: oxidized low-density lipoprotein; ROS: reactive oxygen species; O_2_^∙−^: superoxide anions.

**Figure 2:**
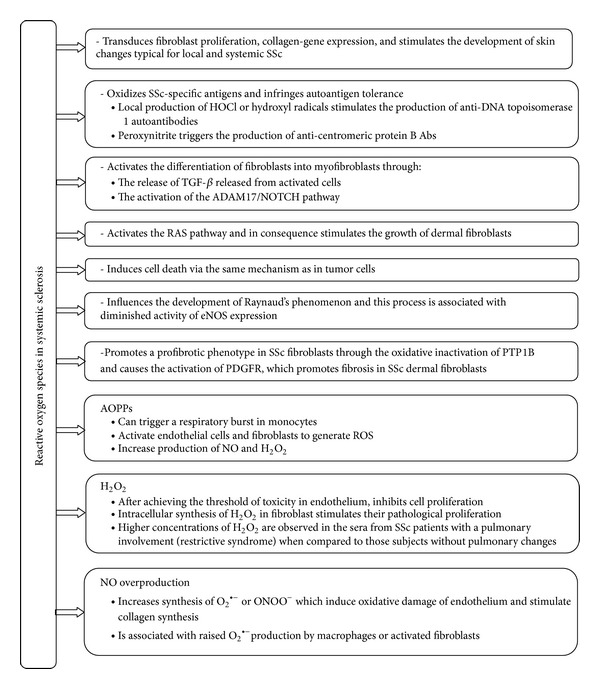
The role of oxidative stress in systemic sclerosis. HOCl: hypochlorous acid; TGF-*β*: transforming growth factor *β*; ADAM17/NOTCH: ADAM17 (disintegrin and metalloproteinase domain-containing protein 17; also known as TACE) involved in the activation of the Notch signaling pathway; Ras proteins: a family of related proteins involved in transmitting signals within cells through the Ras pathway and belonging to a class of proteins called small GTPase; eNOS: endothelial nitric oxide synthase (eNOS or NOS3); PTP1B: protein tyrosine phosphatases 1B; PDGFR: platelet-derived growth factor receptor; AOPPs: advance oxidation protein products; NO: nitric oxide; H_2_O_2_: hydrogen peroxide; O_2_^∙−^: superoxide anions; ONOO^−^: peroxynitrite.

In the development of SSc, many genetic and environmental factors may initiate the onset and stimulate the progression of the disease [5]. However, recently many studies underline the possible role of oxidative stress processes in the pathogenesis of SSc and show a new aspect of this disease (Figure 1) [6–9]. The production of reactive oxygen species (ROS) by skin and visceral fibroblasts as well as endothelial cells has been suggested as a background pathology, mainly in progressive SSc [6]. Synthesized ROS include superoxide anions (O_2_^∙−^), hydrogen peroxide (H_2_O_2_), hydroxyl radicals (^*∙*^OH), and/or peroxynitrite (ONOO^−^) (Figure 2). Indeed, skin and visceral fibroblasts spontaneously produce large amounts of ROS that trigger collagen synthesis [6, 7]. Given the fact that oxidative stress is involved in the pathogenesis of this disease, many trials with antioxidative treatment have been undertaken to prevent proliferation of the fibroblasts and inhibit the progression of SSc [9–20].

In this paper, we summarize the role of oxidative stress processes in the development of various patterns of SSc and describe the mechanisms of action of different prooxidant molecules synthesized by endothelial cells and fibroblasts. We also discuss the efficiency of the physiological antioxidant system in the development and progression of the disease, the possibilities of the beneficial influence of pharmacological and nutritional antioxidants, and putative therapeutic strategies, which could be effective in the supportive treatment of systemic sclerosis.

## 2. The Basic Background Pathology of Oxidative Stress in Systemic Sclerosis 

Oxidative stress processes are one of the main background mechanisms present in many metabolic, cardiovascular, neurodegenerative, and neoplastic diseases and many others [6, 13, 18, 20–23]. Because of the common prevalence of prooxidant activity in the development of various pathologies, scientific curiosity has also focused on systemic sclerosis. Also in the early stages of systemic sclerosis, oxidative stress is considered to be a background factor in the development and activity of this disease [6, 8–11, 20, 24], and it is suggested as contributing to clinical manifestations associated with SSc [6]. In* in vitro* studies, sera from patients with SSc can induce the production of ROS in endothelial cells and in proliferating fibroblasts [9]. Studies on animal models have suggested that oxidative processes can influence the onset and course of this disease [24–27]. The injection of intradermal ROS-generating substances stimulates the development of skin changes typical for local and systemic SSc [24].

Prooxidants may also increase the production of autoantibodies. In animal models and clinical studies, local production of hypochlorous acid (HOCl) or hydroxyl radicals can stimulate the immune response and the production of anti-DNA topoisomerase 1 autoantibodies, whereas the generation of peroxynitrite triggers the production of anticentromeric protein B antibodies [24, 28]. Some studies have even shown that the presence of autoantibodies in endothelial cells and in fibroblasts in SSc is not correlated with serum-induced ROS production or cell proliferation [29, 30]. However, the role of autoantibodies cannot be totally ruled out. The above-mentioned phenomenon is also confirmed by anti-PDGRF antibodies (anti-PDGFR Abs), which can trigger the production of ROS and, thus, activate SSc development [31].

### 2.1. Oxidative Stress in Fibroblastic Cells

Unquestionably, permanent overproduction of ROS stimulates the inflammatory reaction, activates the differentiation of fibroblasts into myofibroblasts [32], stimulates the growth of dermal and visceral fibroblasts [7], causes fibrotic complications [33, 34], and (in high concentrations) can induce cell apoptosis (Figure 2) [35–37].

Fibrosis can be activated by NO and is associated with digital ischemia or pulmonary arterial hypertension (PAH) [5, 38]. The extremely disturbed metabolism and overproduction of NO cause increased synthesis of superoxide anions, O_2_^∙−^, and peroxynitrite, ONOO^−^, which lead to cell injuries and death [6, 37, 39, 40]. NO is largely oxidized to nitrate (NO_3_^−^) and nitrite (NO_2_^−^) and increased plasma ratio NO_3_^−^ */*NO_2_^−^ levels are found [38, 39]. Additionally, an elevated nitration of plasma proteins (measured by ONOO^−^ markers) is found in many organs, including skin fibroblasts [41]. Moreover, in fibrogenesis NO directly stimulates transcription factors (such as NF-*κ*B, SP-1, and AP which inhibit collagen gene expression) [42] and regulates prolyl hydroxylase—an enzyme important in the posttranslational processing of collagen [43].

NO is synthesized from L-arginine by NO synthase (NOS). NOS is expressed in three main isoforms: neuronal (nNOS or NOS1), endothelial (eNOS or NOS3), and inducible (iNOS or NOS2). The last one is present mainly in the inflammatory state of fibroblasts [44, 45]. In sites of active inflammation, the stimulated iNOS isoform is responsible for increased synthesis of NO, which is associated with raised O_2_^∙−^ production by macrophages or activated fibroblasts [46]. With the progression of the disease, the production of iNOS is upregulated in skin fibroblasts [45]. Another synthase—eNOS—also participates in the pathology of SSc and in eNOS knock-out mice decreased bioactivity of NO causes prolonged pulmonary fibrosis [47].

In SSc dermal fibroblasts, collagen synthesis is also promoted by platelet-derived growth factor (PDGF) and its receptor (PDGFR) promotes fibrosis. PDGFR is phosphorylated upon PDGF stimulation and is dephosphorylated by protein tyrosine phosphatases (PTPs), including PTP1B. A study by Tsou et al. proved that ROS may promote a profibrotic phenotype in SSc fibroblasts through the oxidative inactivation of PTP1B, leading to pronounced activation of PDGFR [20]. In fibroblasts isolated from the skin of patients with diffuse SSc, levels of ROS and type I collagen were significantly higher and the amounts of free thiol were significantly lower when compared to normal fibroblasts. The activity of PTP1B was inactivated by the raised levels of ROS in SSc fibroblasts [20]. Moreover, anti-PDGF receptor antibodies [31] and probably also environmental factors [31, 48, 49] could initiate a secondary self-maintained process that is associated with the production of advanced oxidation protein products (AOPPs). The elevated AOPPs concentration can trigger a respiratory burst in monocytes [50, 51]. AOPPs also activate endothelial cells and, to a lesser extent, fibroblasts to generate ROS. They can lead to the production of either NO or H_2_O_2_ in SSc fibroblasts and stimulate their proliferation [6, 7, 9].

Another prooxidative molecule in SSc is 8-isoprostane—a nonenzymatic eicosanoid produced during the random oxidation of tissue phospholipids by oxygen radicals [8, 52]. This is considered a reliable biomarker of oxidative stress and antioxidant deficiency because of its biochemical stability [52]. 8-Isoprostane level increases by 75-fold in SSc patients in both forms, that is, dSSc and lSSc [8]. The serum concentration of this marker correlates inversely with pulmonary function and can be a useful serological marker for the severity of lung fibrosis in SSs [8, 53].

### 2.2. Oxidative Stress in Endothelial Cells

In SSc, oxidative processes cause the activation of and damage to endothelium. The mechanism of endothelial dysfunction is equivocal; however, the development of the vasculopathy includes many serological biomarkers (endothelin, which leads to vessel vasoconstriction), cell adhesion molecules (e.g., E-selectin), antiendothelial antibodies, and ROS (including nitric oxide) [39, 43]. Synthesized ROS selectively activate endothelial cells, leading to vascular complications [33, 34]. In SSc patients, endothelial cells reveal the ability to induce the production of H_2_O_2_. It is suspected that the high level of H_2_O_2_ generated in endothelium probably achieves the threshold of toxicity and thus inhibits intracellular processes [9]. When vascular disorders have been present, inhibited endothelial cell growth associated with increased nitric oxide (NO) overproduction has been observed [9, 38]. However, in SSc a paradoxical decrease in NO production by eNOS in endothelial cells is also observed, and this can be explained by the rapid reaction of NO and O_2_^∙−^ to generate the reactive intermediate ONOO^−^ [6, 39, 41]. Diminished eNOS expression and vasculopathy contribute to Raynaud's phenomenon—a pathology often present in SSc patients [54, 55]. With the progression of the disease, the production of eNOS is downregulated [45].

**Figure 3:**
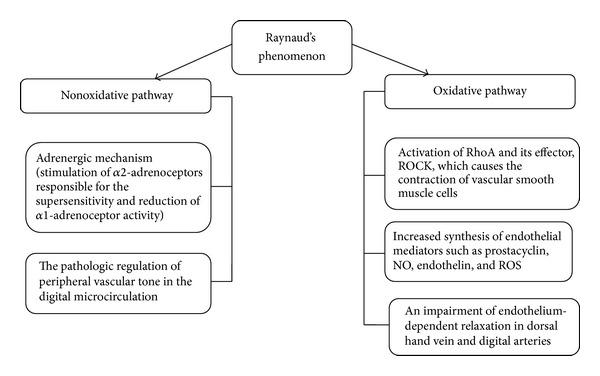
Background pathology of SSc-associated Raynaud's phenomenon. RhoA: Ras homolog gene family, member A (a small GTPase protein regulating the actin cytoskeleton in the formation of stress fibers); ROCK: Rho kinase; NO: nitric oxide, ROS: reactive oxygen species.

Another marker that directly reflects free radical formation in endothelium is 8-isoprostane—a potent vasoconstrictor revealing platelet proaggregant functions and stimulating endothelial cells to bind monocytes [56]. An increased level of isoprostanes correlates with the extent of vascular damage in Raynaud's phenomenon—a primary or secondary condition to SSc [52]. This vasculopathy occurs in more than 90% of patients with systemic sclerosis and results from an excessive, vasoconstrictive response to low temperatures and other stimuli. Many causes have been suggested in the pathogenesis of this vasculopathy (Figure 3). Cutaneous vasoconstriction by direct local cooling induces a response, which is in part adrenergically dependent (nonoxidative pathway). Local cooling causes a temperature-dependent inhibition of basal NOS activity (particularly nNOS and iNOS) [57, 58] associated with hypertrophy and occlusion of the vasculature [59].

Because the presynaptic blockade of noradrenalin release from vasoconstrictor nerve fibers does not completely eliminate the vessel spasm stimulated by low temperatures and the construction of a vascular bed is also observed in the internal organs, it is suggested that other nonadrenergic mechanisms could be involved in this process (oxidative pathway) [60, 61]. The endothelial cells release mediators, such as prostacyclin, NO, and endothelin; increased ROS generation is observed as well [46, 62]. NO acts as a physiological vasodilator and improves peripheral ischemia in SSc, but in Raynaud's phenomenon increased generation of NO during reperfusion injury reacts with superoxides and forms highly reactive hydroxyl radicals, which rapidly diffuse across cell membranes and lead to cell injury [22, 46].

## 3. Antioxidants in Systemic Sclerosis 

### 3.1. Antioxidant Defense Mechanism in SSc

**Figure 4:**
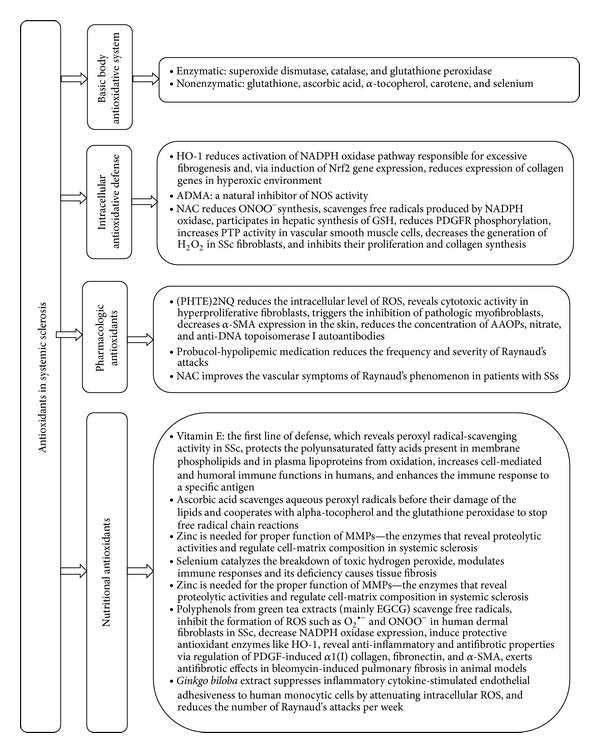
Antioxidative defense in systemic sclerosis. HO-1: haemoxygenase-1, NADPH oxidase: nicotinamide adenine dinucleotide phosphate-oxidase, Nrf2: nuclear factor erythroid-2-related factor 2, ADMA: asymmetric dimethylarginine, NOS: nitric oxide synthase, NAC: N-acetyl-l-cysteine, ONOO^−^: peroxynitrite, GSH: glutathione, PDGFR: antiplatelet derived growth factor receptor, PTP: protein tyrosine phosphatases, H_2_O_2_: hydrogen peroxide, (PHTE)2NQ: 2,3-bis(phenyltellanyl)-naphthoquinone, ROS: reactive oxygen species, *α*-SMA: alpha-smooth muscle actin, AOPPs: advance oxidation protein products, MMPs: matrix metalloproteinase-1, EGCG: (−)-epigallocatechin-3-gallate, O_2_^∙−^: superoxide anions, and PDGF: antiplatelet derived growth factor.

Antioxidative defense involves not only enzymatic antioxidants (superoxide dismutase, catalase, and glutathione peroxidase) but also nonenzymatic molecules (glutathione, ascorbic acid, *α*-tocopherol, carotene, and selenium). These contribute to the primary defense against ROS (Figure 4). Moreover, many antioxidative mechanisms are involved in attenuating intracellular oxidative stress. One of these is nuclear factor erythroid-2-related factor 2 (Nrf2)—the master regulator of inducible antioxidant responses, which can attenuate cellular injury from oxidative stress. Nrf2 is a mediator of the antioxidant response element (ARE) in regulatory regions of several antioxidant enzymes (such as glutathione peroxidase or superoxide dismutase and catalase) [63]. The lack of Nrf2 in animal models (Nrf2−/− knock-out mice) stimulates the expression of extracellular matrix genes such as collagens under a hyperoxic environment [63] and causes increased bleomycin-induced pulmonary fibrosis [64]. Nrf2 has a critical role in protection against pulmonary fibrosis through an enhancement of cellular antioxidant capacity and by the influence on lung Th1/Th2 balance (increased expression of Th2 cytokines, such as interleukin-4 and interleukin-13 in the lungs of Nrf2-deficient mice is observed) [63, 64]. Thus, Nrf2 should be considered a potential factor for antifibrotic therapy in SSc.

Nrf2 influences haemoxygenase-1 (HO-1) activity. HO-1 is the protective antioxidant enzyme induced by NO and Nrf2/HO-1 axis is a major mechanism in antioxidative defense and inflammation [23]. On the other hand, HO-1 is also responsible for physiological oxidative homeostasis inside cells via induction of Nrf2 gene expression (HO-1/Nrf2 axis) [65]. HO-1 can diminish activation of the NADPH oxidase pathway responsible for an increase in collagen synthesis and myofibroblast differentiation in fibrosis [66]. Moreover, cells overexpressing HO-1 are more resistant to oxidant-induced toxicity than controls [21].

Another possible mechanism responsible for reduced production of NO is circulating ADMA—a natural inhibitor of NOS activity [67]. ADMA in the regulatory feedback mechanism causes decreased NO overproduction and the increased activity of this enzyme is present in late stages of SSc (mainly in diffuse SSc) [16, 43].

The molecule, which prevents oxidative damage in SSc, is N-acetyl-l-cysteine (NAC). NAC acts as a precursor for the substrate (l-cysteine) in the synthesis of hepatic glutathione (GSH) and replenishes GSH in deficient cells [68–70]. NAC scavenges free radicals produced by the multicomponent nicotinamide adenine dinucleotide phosphate (NADPH) oxidase (Nox) [68], influences protein thiols, supports glutathione synthesis, and generates free sulfhydryl groups [69]. Moreover, NAC not only decreases cellular O_2_^∙−^ but also restores PTP1B activity along with an improvement of the PDGFR phosphorylation. In consequence, the numbers of tyrosine-phosphorylated proteins are diminished, the level of type I collagen is reduced, and the fibroblast proliferation is decreased [20, 68, 70, 71].

In fibrotic skin disorders, PDGF induces enzymes from Nox complexes, Nox1 and Nox2, while TGF*β* stimulates Nox4 to mediate fibrotic effects [72, 73]. Nox enzymes are the major producers of endogenous ROS by fibroblast and activated leukocytes in the pathogenesis of skin fibrosis [43, 72, 73]. Nox enzymes not only promote fibroblast proliferation and collagen gene expression but also upregulate TGF*β*1, *α*-smooth muscle actin (*α*-SMA, the myofibroblast marker), chemokine ligand-2 (CCL-2, a chemoattractant for monocytes and T lymphocytes), and PDGFR [20, 43, 74–76]. Current understanding of Nox in SSc shows the potential possibilities of Nox-based antifibrotic therapy—a clinical target in the management of skin fibrosis (mainly in dSSc).

### 3.2. Antioxidants as Therapeutic Factors in the Supportive Treatment of SSc

Given the growing evidence of the influence of oxidative stress on the development and progress of systemic sclerosis, antioxidant therapy has been proposed as a supportive therapy in the pharmacological treatment of this disease [10, 11]. The main aim of such therapy is to diminish ROS-induced endothelial damage and vasculopathy. However, there is limited evidence of the positive antioxidative effect of fibrotic processes in systemic sclerosis [13, 16, 26, 27, 68]. Classical antioxidants such as antioxidative vitamins (ascorbic acid, *α*-tocopherol, and *β*-carotene) and minerals (zinc, selenium) have been found in lower concentrations in plasma of patients when compared to healthy controls [10, 11, 77, 78].

#### 3.2.1. Antioxidative Vitamins

Vitamin E, a potent chain-breaking intracellular antioxidant and the first line of defense against lipid peroxidation, has been studied in SSc [12, 78]. Because of its peroxyl radical-scavenging activity, vitamin E protects the polyunsaturated fatty acids present in membrane phospholipids and in plasma lipoproteins. *α*-Tocopherol mainly inhibits the synthesis of newly synthesized ROS, while *γ*-tocopherol traps and neutralizes existing free radicals [12, 79]. Besides its positive influence in attenuating oxidative stress processes, vitamin E stimulates the body's defense, enhances humoral and cell immune responses, and increases phagocytic functions [80, 81]. The supplementation of this vitamin considerably increases cell-mediated and humoral immune functions in humans [81, 82].

Because Raynaud's phenomenon is related to ROS-induced endothelial damage, antioxidative therapy has also been suggested in this pathology [13]. However, some clinical trials have shown limited success in treatment with antioxidants such as *α*-tocopherol or ascorbic acids (these vitamins did not improve microvascular perfusion after cold exposure and did not decrease urinary markers of oxidative stress such as F(2)-isoprostanes) [83, 84].

More promising are the results of the use of vitamin E in pulmonary complications. Ostojic and Damjanov assessed the effects of *α*-tocopherol 400 IU/day (268 mg/day) and ascorbic acid 1,000 mg/day (given through 6 months) on skin thickening and lung function in patients with early diffuse cutaneous SSc. Patients treated with cyclophosphamide associated with antioxidant supplementation have a significantly lower skin thickening progression rate (STPR) when compared to patients only on cyclophosphamide monotherapy [13]. *α*-Tocopherol upregulates the expression of cytosolic phospholipase-A2 and cyclooxygenase-1 and, in consequence, increases the release of prostacyclin, a potent vasodilator and inhibitor of platelet aggregation [85].

The dietary intake of ascorbic acid is also low in SSc patients and this is usually caused by the malabsorption syndrome resulting from increased collagen deposition in the intestines or bacterial overgrowth in SSc patients [86]. Another possible explanation is an altered renal clearance of the aqueous phase of ascorbic acid and its increased excretion in SSc subjects [87].

#### 3.2.2. Antioxidative Minerals

Selenium is an essential part of enzyme glutathione peroxidase (GSH-Px), which catalyzes the breakdown of toxic hydrogen peroxide. Selenium deficiency causes tissue fibrosis [88]. Additionally, selenium reveals the possibility to modulate inflammatory [89] and immune responses [90]. Early studies have shown that the dietary intake of selenium is low in SSc patients, but this is not dependent on a dietary deficiency, but rather malabsorption syndrome [86].

The antioxidant properties of zinc have also been confirmed. Zinc is needed for the proper functioning of MMPs—the enzymes that reveal proteolytic activities and regulate cell-matrix composition in systemic sclerosis [91–93].

#### 3.2.3. Other Nutritional Antioxidants

More recently, natural antioxidants such as polyphenols, which are present in many plant foods, have been studied as possible nutritional factors in decreasing oxidative stress. One of these is (−)-epigallocatechin-3-gallate (EGCG) present in green tea extracts (*Camellia sinensis*), which is effective in eliminating oxidative stress in SSc. EGCG reveals the ability to scavenge free radicals, inhibits the formation of ROS (such as O_2_^∙−^ and ONOO^−^), and reduces oxidative stress [94]. EGCG has a higher potential antioxidant capability than*α*-tocopherol or vitamin C [95] and reduces ROS synthesis through decreasing NADPH oxidase expression [96]. In SSc, EGCG inhibits transcription factors (Nrf2, NF-*κ*B, and AP-1), regulates multiple signal transduction pathways such as MAP kinases, and induces protective antioxidant enzymes such as HO-1 [15, 21, 23]. In animal models, EGCG reveals anti-inflammatory and antifibrotic properties via regulation of PDGF-induced*α*1(I) collagen, fibronectin, and*α*-SMA [26, 27]. In SSc patients and healthy controls, EGCG reduces oxidative stress through inhibition of ROS stimulated by TGF*β* in human dermal fibroblasts [16].

Another plant antioxidant is* Ginkgo biloba* extract (a Chinese herb), which suppresses inflammatory cytokine-stimulated endothelial adhesiveness to human monocytic cells by attenuating intracellular ROS formation [97]. This attenuates the TNF-*α*-induced vascular cell surface and total protein expression of adhesion molecules such as VCAM-1 and ICAM-1 (in SSc patients, increased levels of TNF-*α* in tissue and blood correlate with disease activity) [29, 35]. This beneficial influence of* Ginkgo biloba* may also reduce oxidative stress processes in systemic sclerosis [98] and seems to be effective in reducing the number of Raynaud's attacks per week in patients suffering from Raynaud's phenomenon [99].

#### 3.2.4. Synthetic Antioxidants

Attempts using synthetic antioxidants (such as probucol-hypolipidemic medication) have also been made and have revealed a reduction in the frequency and severity of Raynaud's attacks [100, 101]. However, in a study by Herrick et al., antioxidative therapy was ineffective in limited cutaneous SSc, which could be explained by the late implementation of antioxidants. The authors suggested that the addition of antioxidants at the onset of the disease (before irreversible tissue injury) may give positive results [17].

In many studies, NAC has shown a beneficial influence on SSc, diminishing cellular ROS in fibroblast and replenishing free cellular thiols [6, 9, 18–20]. NAC inhibits fibroblast proliferation and collagen synthesis [6] and reduces peroxynitrite (ONOO^−^) synthesis by activated lung macrophages from SSc patients* in vitro* [68]. Moreover, it potentiates the antiproliferative effect of 5-fluorouracil (5FU)—one of the immunosuppressants used in lung idiopathic interstitial fibrosis [68]. In consequence, fibroblast proliferation is inhibited by 78% [9]. NAC also beneficially influences cultured vascular smooth muscle cells, where it reduces PDGFR phosphorylation and increases protein tyrosine phosphatases (PTP) activity [102]. This improves the vascular symptoms of Raynaud's phenomenon in patients with SSc [14, 18, 19].

## 4. Conclusion

Undeniably, oxidative stress (local and systemic) is one of the important mechanisms which cause intracellular changes and stimulate skin and visceral fibrogenesis. Understanding the molecular mechanism of the oxidative cascade in SSc provides a new insight into this disease. Besides this, assaying serum-induced ROS production allows for an estimation of the clinical activity of SSc, which can be followed by appropriate treatment. Currently, we know that such molecules as probucol, NAC, EGCG, polyfenols, and antioxidative vitamins and minerals may be useful in the supportive therapy of SSc and Raynaud's phenomenon (some results are already very promising).

Although recent findings regarding oxidative stress and supportive antioxidant therapy are very encouraging, further studies are necessary to confirm new mechanisms of oxidative cascades in fibroblasts and define the therapeutic potential of analyzed antioxidants in SSc. This may provide the background for possible antioxidative and pharmacological management, which not only could enable effective treatment but maybe also slow down the progression of the disease. If studies confirm the efficacy of nutritional antioxidants, they could provide the basis for specific nutritional recommendations supportive to pharmacological treatment.

## Abbreviations

ADAM17/NOTCH: Disintegrin and metalloproteinase domain-containing protein 17 involved in the activation of the Notch signaling pathway
ADMA: Asymmetric dimethylarginine
AOPPs: Advance oxidation protein products
ARE: Antioxidant response element
*α*-SMA: *α*-Smooth muscle actin
BAL: Bronchoalveolar lavage
CCL-2: Chemokine ligand-2
dSSc: Diffuse cutaneous systemic sclerosis
EGCG: (−)-Epigallocatechin-3-gallate
eNOS: Endothelial nitric oxide synthase (NOS3)
5FU: 5-Fluorouracil
GSH: Glutathione
GSH-Px: Glutathione peroxidase
H_2_O_2_: Hydrogen peroxide
HOCl: Hypochlorous acid
HO-1: Haemoxygenase-1
iNOS: Inducible nitric oxide synthase (NOS2)
lSSc: Limited cutaneous systemic sclerosis
MMP: Matrix metalloproteinase-1
NAC: N-Acetyl-l-cysteine
NADPH: Nicotinamide adenine dinucleotide phosphate
NOS: Nitric oxide synthase
nNOS: Neuronal nitric oxide synthase
NO: Nitric oxide
NO_2_^−^: Nitrite
NO_3_^−^: Nitrate
NOS: Nitric oxide synthase (NOS1)
Nrf2: Nuclear factor erythroid-2-related factor 2
Nox: NADPH oxidase—nicotinamide adenine dinucleotide phosphate-oxidase
O_2_^∙−^: Superoxide anions
^*∙*^OH: Hydroxyl radicals
ONOO^−^: Peroxynitrite
oxLDL: Oxidized low-density lipoprotein
PDGF: Antiplatelet derived growth factor
PDGFR: Platelet derived growth factor receptor
PRP: Protein tyrosine phosphatases
(PHTE)2NQ: 2,3-Bis (phenyltellanyl)-naphthoquinone
PTP1B: Protein tyrosine phosphatases 1B
PAH: Pulmonary arterial hypertension
Ras proteins: A family of related proteins involved in transmitting signals within cells through the Ras pathway, which belong to a class of proteins called small GTPase
RDAs: Recommended dietary allowances
RhoA: Ras homolog gene family, member A (a small GTPase protein regulating the actin cytoskeleton in the formation of stress fibers)
ROCK: Rho kinase
ROS: Reactive oxygen species
STPR: Skin thickening progression rate
TGF-*β*: Transforming growth factor *β*.
